# Metagenomic assembled plasmids of the human microbiome vary across disease cohorts

**DOI:** 10.1038/s41598-022-13313-y

**Published:** 2022-06-02

**Authors:** S. R. Stockdale, R. S. Harrington, A. N. Shkoporov, E. V. Khokhlova, K. M. Daly, S. A. McDonnell, O. O’Reagan, J. A. Nolan, D. Sheehan, A. Lavelle, L. A. Draper, F. Shanahan, R. P. Ross, C. Hill

**Affiliations:** 1 APC Microbiome Ireland, University College Cork, Co. Cork, Ireland; 2 School of Microbiology, University College Cork, Co. Cork, Ireland; 3 Department of Medicine, University College Cork, Co. Cork, Ireland

**Keywords:** Computational biology and bioinformatics, Microbial genetics, Microbial ecology

## Abstract

We compiled a human metagenome assembled plasmid (MAP) database and interrogated differences across multiple studies that were originally designed to investigate the composition of the human microbiome across various lifestyles, life stages and events. This was performed as plasmids enable bacteria to rapidly expand their functional capacity through mobilisation, yet their contribution to human health and disease is poorly understood. We observed that inter-sample β-diversity differences of plasmid content (plasmidome) could distinguish cohorts across a multitude of conditions. We also show that reduced intra-sample plasmidome α-diversity is consistent amongst patients with inflammatory bowel disease (IBD) and *Clostridioides difficile* infections. We also show that faecal microbiota transplants can restore plasmidome diversity. Overall plasmidome diversity, specific plasmids, and plasmid-encoded functions can all potentially act as biomarkers of IBD or its severity. The human plasmidome is an overlooked facet of the microbiome and should be integrated into investigations regarding the role of the microbiome in promoting health or disease. Including MAP databases in analyses will enable a greater understanding of the roles of plasmid-encoded functions within the gut microbiome and will inform future human metagenome analyses.

## Introduction

Until the development of metagenomic sequencing, many of the microbes residing in the human gut were largely uncharacterised. Over the past two decades, intensive research investigated the human microbiota and has provided evidence that it contributes to health and disease. An emerging dogma is that there is no single combination of microorganisms that define a “healthy” microbiota. Furthermore, microbial community changes that correlate with a disease do not necessarily indicate causation. While bacteria have been the focus of most human microbiome studies, more research is being conducted on the composition and role of archaeal, fungal and viral components^1–3^. It is likely that all of these affect microbiome composition and functionality, and therefore impact on human health.

Plasmids are autonomously replicating DNA elements, present in approximately 50% of bacteria^4^. The majority of characterised bacterial plasmids are circular, but linear and eukaryotic plasmids have also been identified^5,6^. Identifying plasmids within metagenomic datasets and distinguishing them from other mobile genetic elements (MGEs) can be challenging. One group of MGEs are termed ‘phage-like plasmids’ and encode viral structural genes to facilitate the transduction of their genomes between hosts, while replicating within bacteria as a plasmid^7^. The blurred boundary between plasmids and bacteriophages (phages) also creates computational difficulties. A popular program VIRSorter is designed to identify phages in metagenomic data based on the enrichment of viral genes, the enrichment of genes of unknown function, the enrichment of shorter genes, and continuous coding of genes on a particular strand^8^. Some of these features are shared with plasmids. The difficulties associated with identifying and characterising plasmids may partially explain their scarcity within databases, with the curated NCBI RefSeq database (release 203) containing 63,237 bacterial genomes but only 5222 plasmid sequences. As a result the contribution of plasmidomes to the overall function of complex host-associated microbial ecosystems remains poorly understood.

Plasmids are important facilitators of the horizontal gene transfer (HGT) of functions that enable bacteria to rapidly adapt to environmental changes. The most obvious example is the spread of antimicrobial resistance (AMR) genes. The current mortality rate of infections with carbapenem non-susceptible *Klebsiella pneumoniae* ranges from 40 to 50%^9^, with IncF Enterobacteriaceae plasmids responsible for spreading nearly 40% of carbapenemase resistance genes worldwide between 2013 and 2018^10^. Plasmids can also provide bacteria with auxiliary metabolic functions that have been shown to be beneficial to humans. As an example, *Ligilactobacillus salivarius* (formerly *Lactobacillus salivarius*) UCC118 encodes a 242 kb megaplasmid that encodes a bile salt hydrolase, a broad-spectrum antimicrobial bacteriocin, and redox balancing pathways^11^. It seems likely that other plasmids harboured by bacteria in the complex gastrointestinal environmental could contribute positively or negatively to human health.

A recent comparison of rat caecal and rumen plasmidomes demonstrated conserved functional traits, including mobilization and stabilization^12,13^. When rumen plasmidomes were compared with their associated microbiomes it was observed that (1) plasmidomes were more diverse than the microbiome, (2) the average similarity of plasmidomes between animals was lower than that of their microbiomes, (3) the compositions of plasmidomes and microbiomes were closely correlated, and (4) the microbiome and plasmidome both changed with diet^14^. These plasmidome studies clearly indicate niche-adapting functions encoded by plasmids from gut environments, suggesting that the total diversity and functional capabilities of plasmids remains underappreciated in most microbiome studies.

IBD primarily refers to two gastrointestinal conditions, Crohn’s disease (CD) and ulcerative colitis (UC). Overlapping symptoms of active CD and UC include frequent diarrhoea, rectal bleeding, abdominal pain, and fatigue. While CD can occur anywhere along the gastrointestinal tract, UC is localised to the colon. Bacterial changes have been consistently identified in the microbiomes of patients with IBD, with decreases in α-diversity and specific β-diversity compositional alterations. However, recent analyses of human gut viral populations associated with CD and UC have reported conflicting results. In 2015, Norman et al. reported an increase in the diversity of tailed phages^15^. Their approach relied upon existing databases, despite studies demonstrating that novel viruses in samples range from 40 to 90%^16^. A database-independent re-analysis of the Norman et al. study data reported no diversity changes in tailed phages amongst patients with CD or UC, but alterations in the abundance of lytic versus temperate phages^17^. The findings of a UC virome study were similarly under scrutiny recently^18^, as one of the main conclusions regarded the association of a giant Mimivirus with disease (~ 500 nm diameter) despite their protocol using a 220 nm filter to remove bacterial cells^19^. Therefore, beyond bacteria, alterations in different components of the microbiome associated with IBD have not significantly advanced our current understanding of IBD’s complex aetiology and pathogenesis.

Here we assembled a human-associated plasmidome database containing more than a thousand putative plasmid sequences (metagenome assembled plasmids, or MAPs). This database has enabled us to analyse the faecal plasmidomes detected within human microbiome sequencing data across various lifestyles, life stages and events, with a particular focus on patients with IBD. We report consistent α-diversity decreases associated with IBD and *C. difficile* infections (CDI), while plasmidome β-diversity differences are observed across multiple human microbiome studies. Furthermore, we investigate the biological basis for plasmidome differences between healthy controls and patients with CD and UC and demonstrate that plasmidome diversity can be restored in patients with IBD ad CDI through faecal microbiota transplants (FMTs). Finally, we show that plasmidome diversity can distinguish healthy controls and patients with IBD and highlight how plasmids may contribute to a bacterium’s functional capacity and subsequently affect gut health.

## Results

### Characterising a human-associated plasmidome

In order to investigate the human gut plasmidome, a database of human-associated MAPs was constructed. This database included MAPs identified in faecal metagenomes sequenced as part of this study and by data-mining the expansive metagenomic assembly of human microbiomes performed by Pasolli et al.^20^. The final MAP database consists of 1,151 putative plasmids, with 123 sequences originating from the 87 microbiomes of control subjects and patients with IBD. All putative plasmids are (1) circular, (2) contain a gene encoding a plasmid replication (Rep) protein, and (3) are less than 90% identical across 90% of their length (see “Methods”). Additional MAPs within the metagenomic data were likely excluded by these stringent selection criteria.

Our analysis was performed without a small circular molecule-enriching amplification bias such as multiple displacement amplification, yielded a plasmid database with members averaging 29 kb (min. 1 kb, max. 315 kb). Specific plasmid Rep proteins are associated with distinct size ranges, with RepL (PF05732) and Rep3 (PF01051) accounting for the majority of plasmid replication control proteins (Supplementary Fig. 1). There were 87 megaplasmids (> 100 kb) detected, with more than half of the megaplasmids detected (47 of 87) carrying five or more AMR genes. The most common antibiotic resistance mechanisms detected were efflux pumps, with the macB macrolide export gene the most abundant (Supplementary Fig. 2).

**Figure 1:**
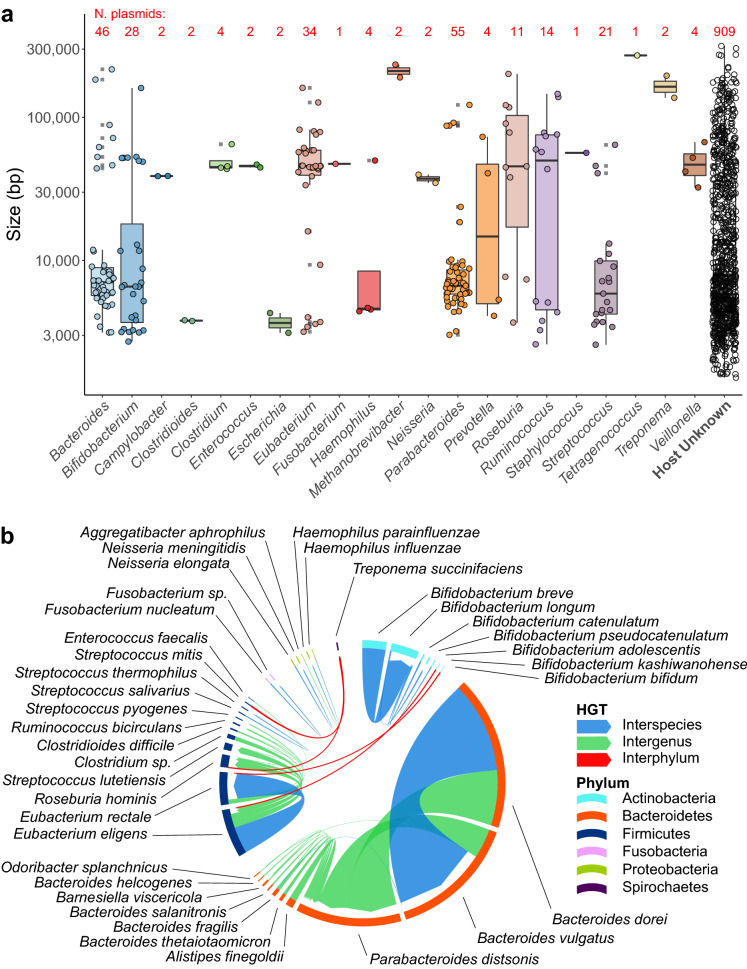
The human gut plasmidome. (**a**) Best predicted bacterial host of plasmids using CRISPR spacers. The number of plasmids per host genus is indicated in red along the top. (**b**) HGT demonstrated by plasmids targeted by CRISPR spacers from two or more bacterial hosts. The outer circle’s colour indicates plasmid source phylum, while the colour and width of arrows indicating type and number of connections between taxa, respectively.

Plasmids are important in the mobilization and rapid adaptation of bacteria to changes in their environment. However, if plasmids enter a naïve bacterial cell they are considered foreign genetic material and can be targeted by host defence mechanisms, such as CRISPR. By identifying bacterial CRISPR spacers targeting MAPs within our database we were able to associate 21% of putative plasmids with a specific bacterium. As expected, the majority of taxa predicted to harbour plasmids are human gut-associated bacteria (Fig. 1a). Where plasmids detected in this study had an NCBI NT database counterpart we compared the accuracy of predicting the host bacterium using CRISPR spacers to those plasmids with a recorded host. Both methods of plasmid host prediction returned similar results (Supplementary Fig. 3), and discrepancies in these host predictions are likely due to the mobile nature and promiscuity of many plasmids.

In several instances, CRISPR spacers targeting a plasmid were detected in two or more hosts, indicating HGT. While multiple interspecies and inter-genus HGT events are observed, inter-phylum HGT was only observed between Actinobacteria (*Bifidobacterium*) and Firmicutes and also between Spirochaetes (*Treponema succinifaciens*) and Firmicutes (Fig. 1b). While Actinobacteria and their plasmids have previously been demonstrated as reservoirs and mobilizers of antimicrobial resistance^21^, *T. succinifaciens* has only been recently described as an enriched gut microbiota taxon found in rural populations^22^.

**Figure 2:**
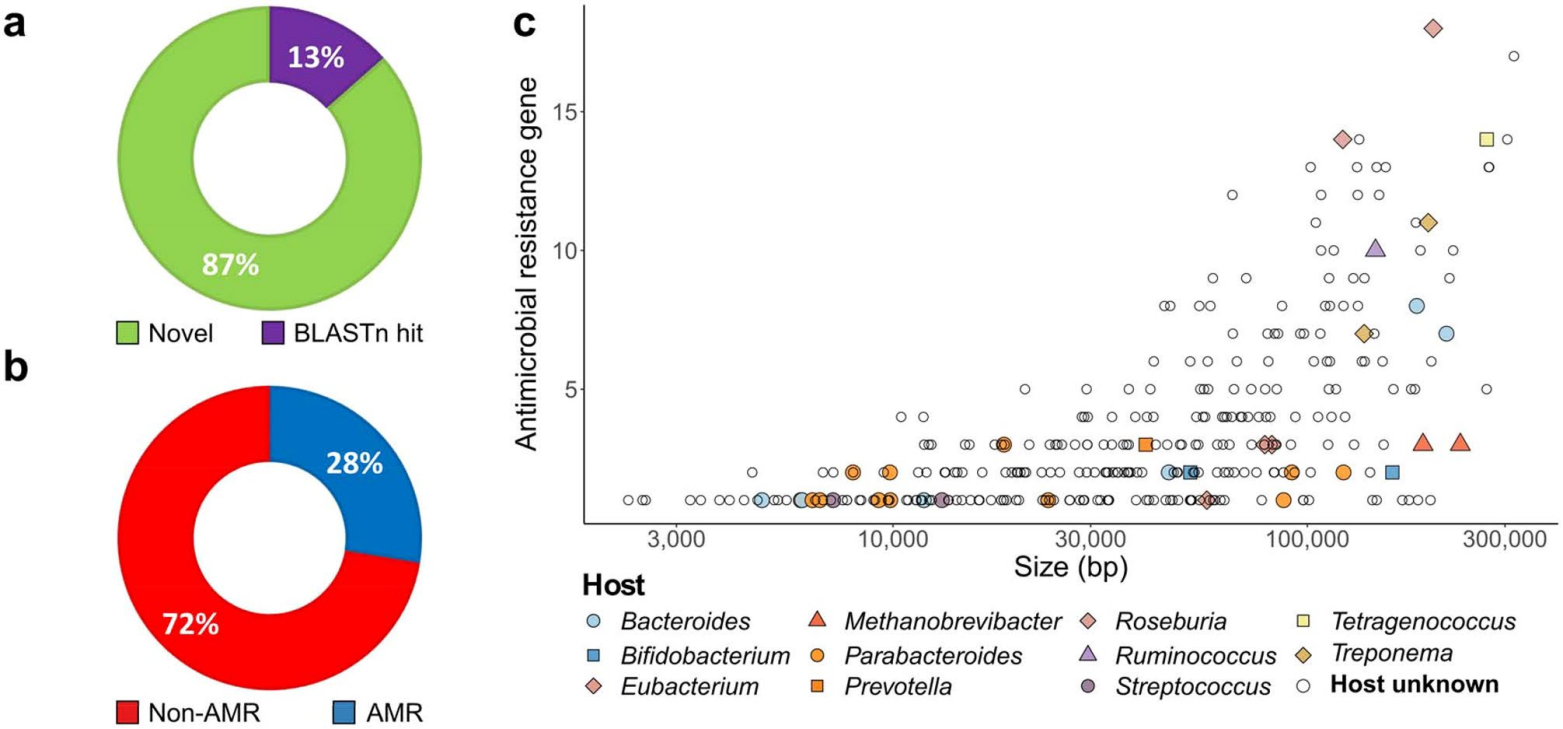
The human plasmidome is an under-explored source of important microbiome functions. The percentage of human microbiome plasmids with (**a**) a nucleotide blast hit against NCBI’s NT database, or (**b**) containing one or more antimicrobial resistance (AMR) genes. (**c**) Plasmid size versus the number of AMR encoding genes, with shape and colour aesthetics highlighting predicted host.

Approximately 86.5% of the human-associated MAPs identified in this analysis are novel in that they are not present in the NCBI NT database, (Fig. 2a; BLASTn E-value threshold: 1E-10). Furthermore, 27.6% of putative plasmids in the human-associated plasmidome database carry one or more AMR gene (Fig. 2b). As plasmid size increases, the capacity to carry more AMR genes similarly increases. While the majority of plasmids could not be assigned to a specific host, megaplasmids (> 100 kb) with multiple AMR genes are spread across bacterial and archaeal taxa (Fig. 2c).

### Investigating plasmid sizes versus their encoded functions

**Figure 3:**
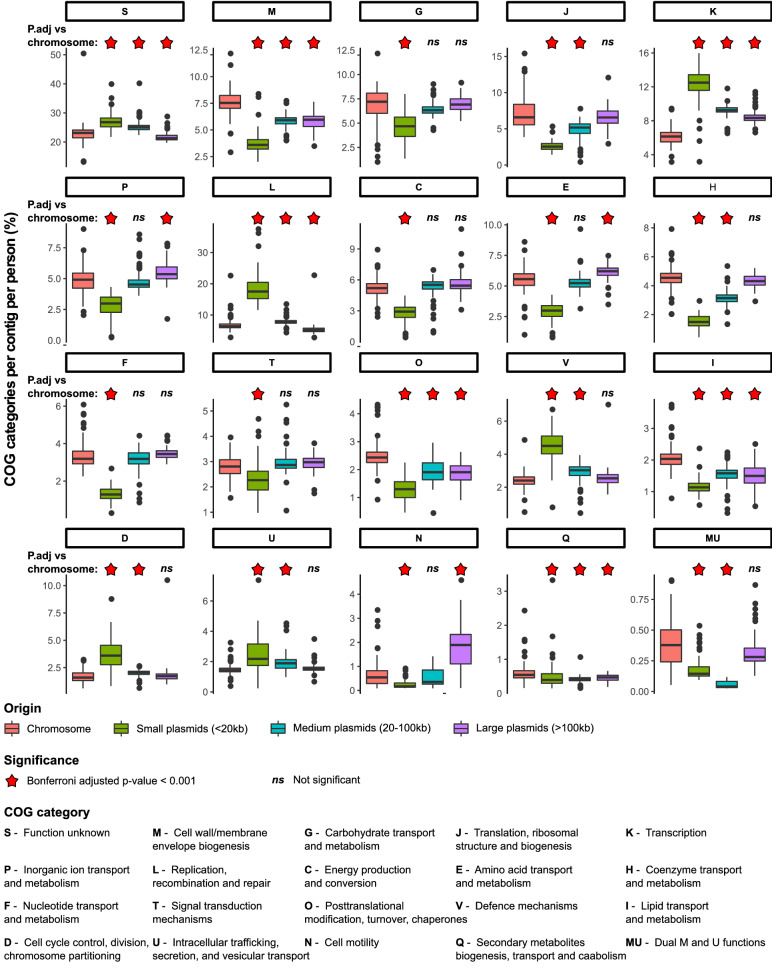
Human microbiome plasmids fulfil ecologically distinct roles. Comparison of the percentage of COG categories per contig encoded on bacterial chromosomes (≥ 100 kb) versus small (≤ 20 kb), medium (20–100 kb), and large (≥ 100 kb) plasmids within human microbiomes.

An analysis of the functions encoded on MAPs of different sizes was conducted as it was expected that maintaining larger plasmids would impose a greater fitness burden on their bacterial host. The percentage of protein-encoding functions categorised into Clusters of Orthologous Gene (COGs) groups for small (< 20 kb), medium (20–100 kb) and large (> 100 kb) plasmids were compared to the overall chromosomal encoded functional predictions (Fig. 3). The top 20 most abundant COG categories are presented by decreasing percentage per contig per person for chromosomal-encoded functions.

As expected, plasmid size influences the number of functions encoded on these mobile genetic elements. However, displaying the abundance of plasmid functions per contig per person demonstrated that smaller plasmids had more proteins of unknown function (category S) while exhibiting a decreased density of proteins playing a role in maintaining the cell wall/membrane, carbohydrate transport, energy production, and amino acid transport (categories M, G, C, and E, respectively; Fig. 3). Additionally, smaller plasmids had the highest densities of genes encoding transcription and defence mechanisms (categories K and V, respectively).

Conversely, the density of COG category functions encoded by large plasmids were more altruistic towards their bacterial hosts (i.e. potentially contributing to their host’s replication and fitness and not merely their own). There were comparable COG densities between large plasmids and chromosomes for carbohydrate transport, translation, energy production, coenzyme metabolism, nucleotide metabolism, or signal transduction mechanisms (categories G, J, C, H, F, and T, respectively). However, an increased density in inorganic ion metabolism, amino acid metabolism, and cell motility are observed amongst large plasmids compared to chromosomes (categories P, E, and N, respectively; Fig. 3).

### Analysing the diversity of human faecal plasmidomes

Differences in the diversity of plasmidomes were assessed across a range of studies that were originally designed to investigate associations between the human microbiome and health. The α-diversity of faecal plasmidomes was not found to differ between; (a) women with or without gestational diabetes, (b) new-borns delivered by spontaneous vaginal delivery or caesarean section, (c) breast-fed or bottle-fed infants, (d) athletes and adults with high and low BMIs, (e) individuals residing in urban or rural environments, (f) controls and patients with colorectal cancer, and (g) controls and patients with Parkinson’s disease (Supplementary Fig. 4). However, a decrease in plasmidome α-diversity was observed with increasing age, reaching statistical significance between young adults and semi-supercentenarians (ages 22–48 and 105–109, respectively; Supplementary Fig. 4h).

**Figure 4:**
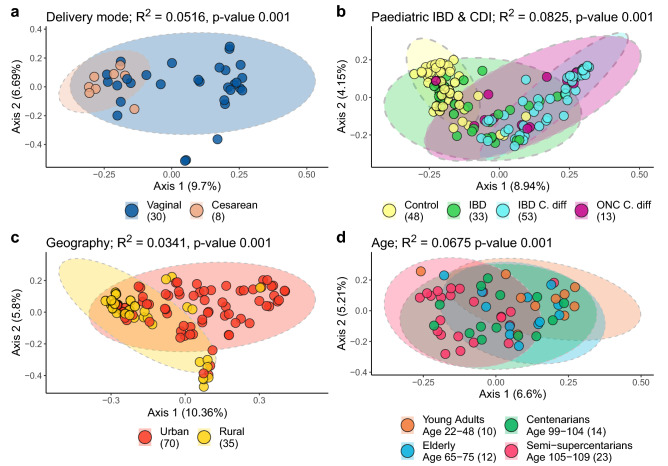
Inter-sample plasmidome differences are widespread across human metagenomic studies. Re-analysis of publicly available metagenomic data originally investigating microbiome differences associated with (**a**) birth mode, (**b**) paediatric IBD and CDI, (**c**) urban versus rural habitation, and (**d**) old age. Beta-diversity analysis was performed using PCoA ordination of Canberra distances. Image panel titles indicate variance (R^2^) and p-values for permutational multivariate analysis of variance (PERMANOVA) tests, with the number of samples analysed per cohort indicated within the brackets of each panel legend.

While plasmidomes α-diversities were seldom different between cohorts, between-sample plasmidome β-diversities varied significantly across the human microbiome studies tested (Supplementary Table 4). For example, the β-diversities differed between new-borns by delivery mode (Fig. 4a), controls and children with IBD and/or CDI (Fig. 4b), urban versus rural geographical residence (Fig. 4c), and increasing age (Fig. 4d). These inter-sample compositional differences observed between human gut plasmidomes reflects the overall differences observed between microbiomes, with positive correlations between whole genome sequencing microbial composition and the predicted plasmid hosts (Supplementary Fig. 5). We observed strong correlations between the relative abundances of *Fusobacterium*, *Methanobrevibacter*, and *Prevotella* and the relative abundance of plasmids predicted to reside in these genera (correlation coefficients: 0.9997, 0.9848, and 0.8687, respectively).

### Human plasmidome alterations are associated with IBD

**Figure 5:**
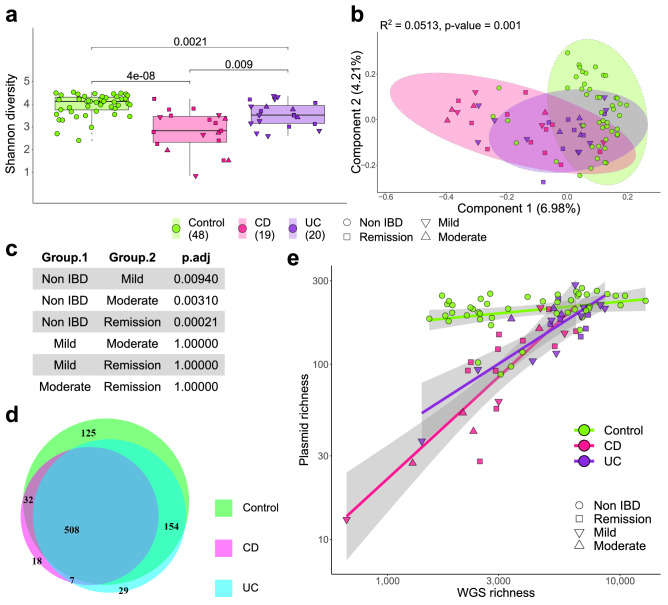
Intra-sample plasmidome differences are associated with IBD. (**a**) Shannon index α-diversity comparisons, and (**b**) β-diversity PCoA ordination of Canberra distances, of control, CD, and UC plasmidomes. The shape aesthetic indicates the recorded severity of patients with IBD, with the number of samples analysed per cohort displayed in brackets within the legend. (**c**) Wilcoxon signed-rank test with Bonferroni-correction to assess statistical significance of plasmidome α-diversity differences between non-IBD controls and patients with IBD grouped by disease severity. (**d**) Proportional Venn diagram of human plasmidome database plasmids detected across the total control, CD, and UC cohorts. The majority of plasmids are detected within all three cohorts (508 of 873 plasmids), with only 18 and 29 plasmids unique to CD and UC, respectively. (**e**) Scatter plot of plasmid richness compared to the overall total microbiome sample richness. The shape aesthetic highlights the reported severity of patients with IBD, with the shading around the linear model regression line representing a 0.95 confidence interval.

A consistent decrease in plasmidome α-diversity was observed between controls and patients with IBD, stratified into ulcerative colitis (UC) and Crohn’s disease (CD), using data from this study and two additional independent IBD studies (Fig. 5a, Supplementary Fig. 6). IBD plasmidome α-diversity differences are also noticeable when Evenness and Richness measures are examined (Supplementary Fig. 7). With regard α- and β-diversity, alterations in the plasmidome appears more pronounced in CD than in UC, relative to controls (Fig. 5a,b). While β-diversity separation of plasmidomes is not driven by disease severity (PERMANOVA p-value 0.669), plasmidome α-diversity can distinguish between controls and patients with IBD regardless of disease severity (Fig. 5c). However, no specific trend was observed between patients recorded with mild or moderate symptoms, nor were there differences between patients with active IBD or within remission.

An analysis of plasmids shared by the control, CD, and UC cohorts indicate many human gut plasmids are conserved between microbiomes. The data generated in this study indicates that 446 plasmids are shared by ten or more individuals irrespective of IBD status, while 141 plasmids are shared in 45 of the 87 individuals tested (see “Supplementary Data”). Furthermore, IBD is not associated with a unique plasmidome, and individually, patients with CD or UC have lost plasmids. The total plasmids present in any of the control, CD, or UC subjects indicate that the majority of plasmids (508 of 873) were present in one or more person from each cohort (Fig. 5d). While control plasmidomes contained 125 unique plasmids, CD and UC cohorts only contained 18 and 29 unique plasmids, respectively. This is also observed in the Richness of control, CD, and UC plasmidomes as the number of plasmids detected within a subject’s faecal sample increases with the overall total microbiome Richness (Fig. 5e). When all samples are considered, there is a moderate but significant correlation between plasmid and total microbiome Richness (Pearson’s correlation: ρ 0.49, p-value 1.34e − 06). Yet when only CD samples are considered, a strong positive correlation is observed (Pearson’s correlation: ρ 0.79, p-value 5.49e − 05).

### Plasmids and plasmidome functions are potential biomarkers of gut health

**Figure 6:**
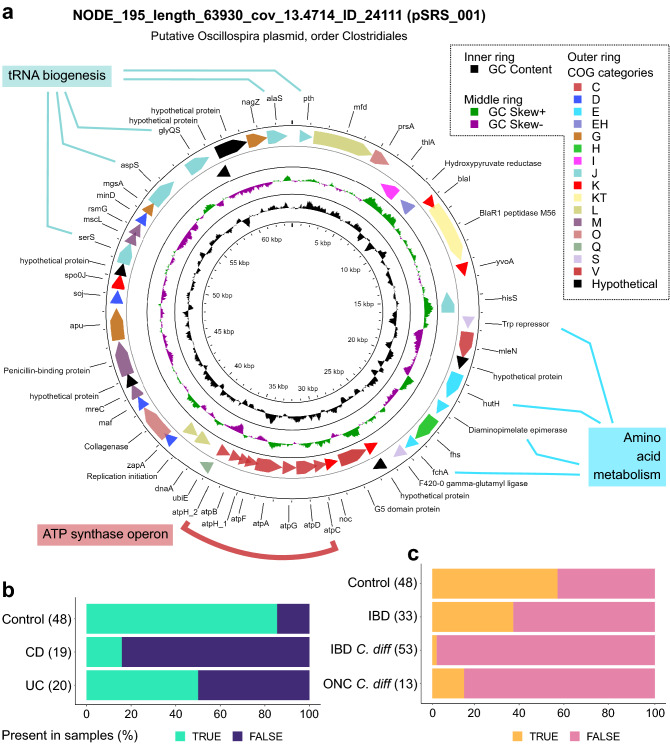
Human microbiome plasmids as biomarkers of gut health. (**a**) Genetic map of putative *Oscillospira* plasmid, NODE_195_length_63930_cov_13.4714_ID_24111 (pSRS_001). In addition to diverse functions, pSRS_001 encodes amino acid synthesis and metabolism genes, and a full F-type ATP synthase operon. Presence of plasmid pSRS_001 in the microbiome of (**b**) adult control, CD, and UC cohorts, and (**c**) paediatric control, IBD, IBD with *C. difficile*, and malignancy (ONC) with *C. difficile* cohorts.

MAPs and their encoded functions were investigated as biomarkers of gut health between controls and patients with IBD. As functions encoded by putative plasmids cover a wide variety of cellular functions, only statistically significant functions after Bonferroni correction are presented (Supplementary Table 5). Of particular interest was the identification of a putative plasmid NODE_195_length_63930_cov_13.4714_ID_24111 (henceforth termed pSRS_001) that could act as a biomarker of IBD (Fig. 6a). The functions of pSRS_001 were predicted using both Prokka and EggNOG, with the closest-related EggNOG functional homologues frequently observed in Oscillospiracaea (order Clostridiales). *Oscillospira* are common gut microbiota members that are predicted to be involved in the degradation of plant-based dietary components^23,24^ and are associated with low BMI and decreased bowel movement frequency^25^.

Amongst other functions, pSRS_001 appears to encode a full ATP synthase operon, genes for tRNA biogenesis, and amino acid metabolism. Many of the functions encoded on pSRS_001 were differentially abundant as the frequency of plasmid detection is higher amongst control plasmidomes (85.4%), while less abundant in patients with CD or UC (15.8% and 50%, respectively; Chi-square test, p-value 3.67E − 07; Fig. 6b). Within the Bushman and colleagues study of paediatric IBD and CDI^26^, which similarly performed Illumina-type sequencing, pSRS_001 was also more abundant amongst controls than children with IBD and CDI (56.3% versus 1.9%, respectively; Fig. 6c).

**Figure 7:**
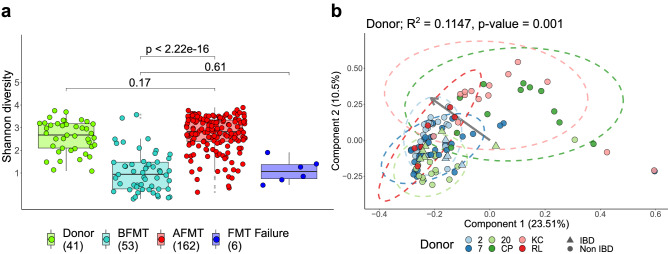
Restoration of the human plasmidome following faecal microbiota transplants (FMT). (**a**) The Shannon index α-diversity of FMT patient plasmidomes after FMT (AFMT) resembles that of donor plasmidomes. No significant difference (Wilcoxon test, p ≤ 0.05) is observed between plasmidome α-diversities before FMT (BFMT) or if the FMT was recorded as unsuccessful in treating *Clostridioides difficile*. (**b**) The β-diversity ordination of faecal plasmidomes after FMT, with the permutational multivariate analysis of variance (PERMANOVA) R^2^ highlighting recipients closely resemble their donors up to 12 weeks AFMT. The origin-centred grey arrow indicates the directionality of increasing intra-sample α-diversities.

Finally, we investigated the impact of faecal microbiota transplants (FMT) on the gut plasmidome. Examining the plasmidomes of donors and recipients confirmed that plasmidome diversity can be restored through FMT (Fig. 7a). No statistical difference was observed between the plasmidome α-diversities of donor and successful FMT recipients, or individuals before or after a failed FMT treatment. A limited number of patients undergoing FMT treatment for CDI also suffer with IBD. Their plasmidome α-diversity increased following FMT and was still elevated after 12 weeks (Supplementary Fig. 8). Additionally, patients treated for CDI had plasmidome compositions similar to their donors after FMT (Fig. 7b; PERMANOVA R^2^ 0.12, p-value 0.001).

## Discussion

Numerous studies have investigated the relationship between humans and their microbiome. However, the plasmids harboured by human-associated bacteria are frequently overlooked. This is despite the clear importance of plasmids in the rapid mobilisation of auxiliary metabolic functions of benefit to bacteria, such as antimicrobial resistance. In this study, we built a human-associated plasmid database in order to leverage new and existing human microbiome data to investigate the human plasmidome and its potential contribution to health or disease. We demonstrate there are clear plasmidome differences within previous human microbiome studies that have been overlooked.

We frequently observed inter-sample plasmidome β-diversity differences between cohorts. This is in agreement with the overall bacteriome compositional differences that are observed during microbiome analyses, as the detection of many plasmids positively correlates with microbial taxa. Specifically, we demonstrate plasmidome β-diversity differences are detectable across studies looking at infant birth mode, early-life diet, paediatric and adult bowel diseases, exercise, rural versus urban lifestyles, and aging. However, demonstrating how these plasmidome differences affect their microbiome’s structure and function requires further attention.

As plasmids are extrachromosomal elements that potentially represent a metabolic burden on their host, we investigated how larger plasmids could provide an ecological benefit to their host bacterium. Indeed, we observed that the density of altruistic energy production and metabolic functions increased amongst larger plasmids, likely offsetting their burden to their host. In contrast, smaller plasmids were enriched in selfish replication and maintenance strategies, including defence mechanisms. Considering the mobile nature of plasmids, the composition and encoded functions of small and large plasmids within a microbiome at specific times could have a profound effect on the phenotypic properties of the bacteria they replicate within. Therefore, additional investigations of plasmidomes will be important to advance a holistic understanding of microbiomes and their association with human health and disease.

We chose to conduct a more in-depth analysis of plasmidome differences associated with IBD, as both plasmidome α-diversity and β-diversity alterations were consistently detected across three independent studies conducted by three research centres. The repeated observation of a decrease in plasmidome α-diversity is consistent with intra-sample decreases in bacterial α-diversity previously noted with IBD^27–31^. Additionally, decreased plasmidome α-diversity was observed amongst patients with CDI, which is also linked to microbiome diversity disruption and frequently associated with IBD^32^. As a validation that plasmidome α-diversity differences are not associated with additional lifestyles, life stages or events, we examined eight publicly available non-IBD datasets where faecal microbiomes were examined as a potential contributor to health or disease. We did not observe plasmidome α-diversity differences in individuals with: (a) gestational diabetes, (b) infants by birth-mode, (c) infant by breast- or bottle-feeding, (d) exercise and body mass index, (e) urban or rural habitation, (f) colorectal cancer, or (g) Parkinson’s disease. However, a decrease in plasmidome α-diversity was observed amongst individuals aged 105–109 years old. Therefore, alterations in plasmidome α-diversity are not widespread, but are a biological feature of IBD and *Clostridioides difficile* infections.

Both α-diversity and β-diversity differences between control and CD and UC plasmidomes can be attributed to plasmid loss. When all cohort members are considered, the majority of plasmids are shared and few plasmids are unique to a specific group. Additionally, as the number of species (Richness) increases within the human microbiome, a concomitant increase in plasmidome richness was observed. The magnitude of change between the overall microbiome and plasmidome is most pronounced for CD, where cohort members had the overall lowest diversities. A clearly significant difference in plasmidome α-diversities is observed between patients with IBD and controls, across both adult and paediatric IBD studies. Interestingly, statistical significance is still evident in α-diversities between patients reporting remission and mild or moderate symptoms, indicating plasmidome α-diversity alone does not explain the aetiology of IBD.

Patients with IBD are at increased risk of developing CDI^33^. Reductions in plasmidome α-diversities were observed in both adults and children suffering with CDI. FMTs have been successfully administered to restore microbiome diversity and treat CDI, while they are currently under investigation for treating IBD^34^. In this study, we demonstrate that the donor plasmidome is transferred during FMT and increases the recipient’s α-diversity. Although limited in terms of the number of patients analysed, the plasmidome α-diversity similarly increased amongst patients with IBD treated for CDI. Understanding the complete microbiome of FMT donors, including their plasmidome, is particularly important as plasmids frequently encode AMR genes and serious adverse events including death have occurred following FMTs^35^.

Numerous plasmid-encoded functions are significantly different between controls and patients with CD and UC. Plasmid-encoded functions fulfil a wide variety of roles, from energy production to drug-resistance and horizontal gene transfer to replication-cycle control. Despite the importance of plasmids in disseminating AMR genes, no specific increase in any antibiotic resistance pathway was observed. However, this may be as a result of the specific cohort analysed, as only four control and one patient with CD were recorded as recently using antibiotics. It would be interesting to investigate plasmidomes in patients using antibiotics in the future, as significant differences in plasmid-encoded AMR genes would be expected.

While the α-diversity of plasmidomes can distinguish control subjects from CD and UC patients, it appears to be a poor indicator of disease severity. However, during analysis of our adult IBD cohort, a putative plasmid common to multiple plasmidomes was frequently encountered. Plasmid pSRS_001 was similarly abundant in a geographically independent study of childhood microbiomes, where it was decreased amongst paediatric IBD and CDI cohorts. Therefore, in conjunction to plasmidome α-diversities, future studies may determine if the presence, absence, or quantitative differences, of specific plasmids can act as a biomarker of IBD. There is currently no cure for CD or UC; therefore, understanding the human microbiome as an aetiological trigger of IBD or developing it into a non-invasive test to manage this debilitating disease represents an important challenge.

## Conclusions

While microbiomes are frequently investigated for their associations with human health and disease, our understanding of specific microbial components and their functions is lacking. Alterations in bacterial diversity are frequently reported within IBD and CDI studies, but we show that there are additional human plasmidome differences that have been neglected as a potential contributor to health and disease and in our overall understanding of the host-associated microbiome. For the first time, we clearly demonstrate plasmid loss is associated with IBD and that this biological feature is more pronounced in patients with CD than UC. As plasmids encode auxiliary functions necessary for niche adaptation and bacterial competitiveness, their loss may partially contribute to the increased microbiome fluctuations observed amongst patients with IBD^30^. While plasmidome diversity alone did not serve as a good biomarker of IBD severity, we show that it was restored in patients administered FMTs to treat CDI. Combined with the identification of a putative plasmid frequently associated with controls and diminished amongst patients with IBD or CDI, we suggest that a greater understanding of the human gut plasmidome may open new avenues for microbiome-based therapeutics, diagnostics, and interventions.

## Methods

### Study recruitment and faecal microbiome sequencing

Consenting adult volunteers were recruited to study APC055, which was approved by the Cork Research Ethics Committee. Relevant clinical data and patient characteristics were recorded for recruited patients. Faecal samples were collected from volunteers without additives or preservatives, transported to the research facility at ambient temperature, and were stored at − 80 °C until processed. To extract whole microbiome DNA, samples were thawed on ice and processed using a QIAamp Fast DNA Stool kit (QIAGEN) as previously described^36^. The quality and concentration of DNA extractions were assessed using the Qubit dsDNA BR kit (ThermoFisher Scientific). Sequencing libraries were prepared following the TruSeq Nano DNA Library Preparation kit, pooled at equivalent concentrations, and samples were sequenced by GATC Biotech AG, Germany on an Illumina HiSeq 2500. A mean of 13.7 M raw microbiome reads were obtained across the 87 whole genome sequencing (WGS) samples processed (min. 1.7 M, max. 31 M).

### Microbiome sequencing of an IBD cohort

We performed total faecal microbiome sequencing of 48 control, 19 CD, and 20 UC volunteers. All patients were recruited from a clinic dedicated exclusively to IBD and managed by an experienced physician where extensive metadata could be collected. Study population data includes basic details (e.g. sex, age), physical attributes (e.g. weight, height), and lifestyle choices (e.g. smoking, alcohol). Additionally, clinical data for patients with IBD includes disease severity, distribution, and activity indices (e.g. Harvey Bradshaw Index, Powell-Tuck Index), age of onset, IBD-specific and generic medications, and surgical history. Summaries of study participant details are provided as Supplementary Tables 1–3, whereas the full detailed metadata resource for all volunteers is available within “Supplementary Data”.

### Sequence read processing

Illumina sequencing reads generated during this study or downloaded from NCBI were processed identically. Adaptor and poor-quality bases were removed using Trimmomatic in paired-end mode (version 0.36^37^). Sequences were trimmed when the Phred quality score dropped below 30 across a 4 bp sliding window, with sequences shorter than 50 bp after processing discarded. NCBI BioProjects downloaded were: PRJNA46321, “Metagenomic Analysis of the Structure and Function of the Human Gut Microbiota in Crohn’s Disease”^38^; PRJNA562600, “Multi-omic analysis of *Clostridioides difficile* infection and response to therapy in pediatric inflammatory bowel disease”^26^; PRJEB15388, “The Diet and Exercise-Microbiome Paradigm: Distinct Functional Profiles of the Athlete Microbiome Revealed by Metagenomic and Metabolomic Analysis”^39^; PRJEB7774, “Gut microbiome development along the colorectal adenoma-carcinoma sequence”^40^; PRJNA553191, “Shotgun metagenomics of human gut microbiota up to extreme longevity and the increasing role of xenobiotics degradation”^41^; PRJNA564397, “Metagenomics Reveals Impact of Geography, Antibiotics and Acute Diarrhoeal Disease on the Gut Microbiome of Central Indian Populations”^42^; PRJNA322188, “Maturation of the Infant Microbiome Community Structure and Function Across Multiple Body Sites”^43^; PRJNA542703, “Whole genome metagenomic analysis of the gut microbiome of differently fed infants”^44^, PRJNA626004, “Shotgun Metagenomics Fecal Microbiome Dysbiosis in Parkinson’s Disease”^45^; and PRJNA406958, “Effect of Oral Capsule– vs Colonoscopy-Delivered Fecal Microbiota Transplantation on Recurrent *Clostridium difficile* Infection: A Randomized Clinical Trial”^46^.

### Building the plasmid database

Sequencing reads of control and IBD microbiomes generated as part of this study were assembled with metaSPAdes (version 3.11.1^47^). Assembled sequences less than 1000 bp were discarded. Protein-coding sequences were predicted using Prodigal (version 2.6.3^48^), with the ‘meta’ option enabled for shorter sequences and Shine-Dalgarno training disabled for reproducibility. Plasmid replication full protein family alignments were downloaded from Pfam (version 32.0); Rep_1 (PF01446), Rep_2 (PF01719), Rep_3 (PF01051), RepL (PF05732), RepA_N (PF06970), and RepA_C (PF04796). Profile hidden Markov models (HMMs) were built and concatenated using HMMER (version 3.1b1^49^). Subsequently, the protein predictions of control and IBD microbiomes were searched using the plasmid replication HMMs. Contigs containing one-or-more plasmid replication proteins were tested for circularity, identified by homology between the contig’s termini. A plasmid search was also conducted against the metagenomic assembly data generated by Pasolli et al.^20^. Once more, the HMM-based plasmid replication protein search followed by circularity detection was employed to identify plasmids.

For all putative plasmids identified, a counter-selection against viral-like sequences was implemented. Briefly, putative plasmids were removed that: (1) had a significant hit against the viral RefSeq database (version 87), (2) had a significant hit against crAss-like phages^50^, (3) were assigned to the *Microviridae* viral family using Demovir (https://github.com/feargalr/Demovir), and (4) were abundant in viral proteins (5 or more prokaryotic viral orthologous groups per 10 kb^51^). Plasmids detected, either from assemblies of this study or the Pasolli et al*.* study, were made non-redundant by removing the smaller of two homologous sequences when the BLASTn identity exceeded 90% across 90% of the smaller sequences’ length. Processed sequencing reads were mapped onto the non-redundant plasmid database using Bowtie2 (version 2.3.4.1^52^), with the number and breadth-of-coverage of reads across contigs calculated using SAMtools (version 1.7^53^) and BEDTools (version 2.26.0^54^), respectively.

### Compiling the CRISPR spacer database

The Pasolli et al*.* metagenomic assembly data was additionally explored for CRISPR spacer sequences against MGEs using PILER-CR^55^. However, in order to run the PILER-CR CRISPR detecting program locally, the Pasolli multi-fasta assembly data files were split into 0.5 Gb files. It was necessary to add a greater-than (“>”) symbol before the first character of the first line of newly generated sequence files after splitting. PILER-CR was looped through all mutli-fasta files, generating files for both CRISPR repeat and CRISPR spacer sequences. The PILER-CR text file outputs were manipulated using ‘awk’ and ‘sed’ commands in unix to obtain CRISPR spacers associated with the name of their original contig. Subsequently, multiple CRISPR spacers from a single assembled contig were sequentially numbered. The predicted taxonomy of contigs yielding CRISPR spacers were assigned through BBMap’s SendSketch function (Bushnell B. sourceforge.net/projects/bbmap/), which is a rapid alignment-free method of comparing genome kmers against JGI’s taxonomy server. A significant BLASTn hit between a detected CRISPR spacer and a plasmid was set at E-value 0.1, due to the short nature of the CRISPR spacer sequences. Additionally, CRISPR spacer hits containing more than 2 nucleotide mismatches or more than a single nucleotide insertion/deletion were discarded as insignificant.

### Bioinformatic and statistical analyses

The following procedures were performed to limit technical biases. Firstly, the Illumina sequencing data from all studies, our own and publicly available, were downloaded and processed identically. The only exception was the human microbiome project (HMP) IBD study, which was generated through 454 sequencing technology. Secondly, to control for the large discrepancies observed amongst plasmid lengths, the number of reads aligning to each sequence was corrected using the reads per kilobase per million reads (RPKM) approach commonly employed in transcriptomic studies. Therefore, we strongly believe the cross-study comparisons and observations of this study are biologically significant rather than technical artefacts.

All bioinformatic analyses were performed in R (version 3.6.1^56^), implemented through R Studio. Sequencing reads mapping to plasmids were considered significant, and not simply aligning to a conserved domain, if 5 or more reads aligned end-to-end against a plasmid and 10% or more of the plasmid’s total length was covered by reads. Read counts were normalised per sample using the reads per kilobase per million sequencing reads procedure (RPKM), and cast into a matrix using the R package “reshape2”^57^. Within sample α-diversity measures were calculated using the R package “vegan”^58^, while the between sample β-diversities were determined through the R package “phyloseq”^59^. β-Diversities shown are principal co-ordinate analysis (PCoA) of Canberra distances, unless otherwise stated. Permutational multivariate analysis of variance (PERMANOVA) statistical tests were calculated using the adonis function of vegan.

Independence between two variables was established using the Chi-square test, or the Fisher’s exact test where 5 or less samples per variable are assessed. The Wilcoxon test was employed to determine statistical difference between two groups, while Kruskal–Wallis compared three or more groups. Where relevant, statistical comparisons were performed prior to performing logarithmic transformations of zero-value containing data. Insertion of the p-values from the Wilcoxon and Kruskal–Wallis non-parametric tests within images was performed using the R package “ggpubr”^60^. Pearson and Spearman correlations were implemented through the cor.test function of the “stats” R package.

Publication quality images were generated using the R package “ggplot2”^61^. All boxplots were produced with default parameters, where the lower and upper hinges represent the first and third quartiles, and whiskers extend 1.5× the interquartile range. Outliers beyond the tips of the upper and lower whiskers were depicted as dark-grey squares for ease of identification. The circular chord diagram was generated using the R packages “circlize” and “chorddiag”^62,63^. Differential abundance analysis of plasmids was performed using the R package “DESeq2”^64^. Proportionally scaled Venn diagrams were generated online with BioVenn^65^. The circular genome map of plasmid pSRS_001 was generated using CGView (Server Beta^66^), with annotations from Prokka^67^ and EggNOG-mapper (v2^68^). The functional predictions of the complete plasmid database was generated by EggNOG-mapper only. Antibiotic resistance encoding genes were specifically detected through a protein BLAST search against the comprehensive antibiotic resistance database (CARD; v3.0.9)^69^. Clustering of homologous nucleotide sequences to identify overall changes in plasmidome functions was performed using CD-HIT, with a 70% identity threshold and word size of 5^70^.

### Ethics approval and consent to participate

Patients with IBD were recruited to donate faecal samples for microbiome analysis through a speciality IBD clinic, run by an experienced physician. Control subjects were enrolled in study protocol APC055, which was approved by the Clinical Research Ethics Committee of the Cork Teaching Hospitals. All methods were carried out in accordance with relevant guidelines and regulations. Informed consent was obtained from all adult donors with a written questionnaire completed to demonstrate their willingness to partake in the study.

## Supplementary Information

Supplementary Information 1.

Supplementary Information 2.

## Data Availability

Additional analyses supporting the conclusions of this study have been supplied as Supplementary Material. The data and scripts required to generate the images and interpret the results of this study are provided as Supplementary Data. The whole genome sequencing data analysed in this study is available through NCBI BioProject code: PRJNA813736.

## Abbreviations

Plasmidome: Plasmid metagenome
IBD: Inflammatory bowel disease
CD: Crohn’s disease
UC: Ulcerative colitis
MGE: Mobile genetic element
HGT: Horizontal gene transfer
AMR: Antimicrobial resistance
FMT: Faecal microbiota transplant
CDI: *Clostridioides difficile* Infection
