# Supplementary material for: Metagenomic assembled plasmids of the human microbiome vary across disease cohorts
Source: Sci Rep. 2022 Jun 2;12:9212. doi: 10.1038/s41598-022-13313-y (PMC9163076; doi:10.1038/s41598-022-13313-y)
Supplement: Supplementary file 1 — Supplementary Information 1. [file 41598_2022_13313_MOESM1_ESM.docx]

**Metagenomic assembled plasmids of the human microbiome vary across disease cohorts**

**Supplementary Material**

Stockdale, *et al.*

Provided are a series of results to support the main conclusions of Stockdale and colleagues. The methods related to the Supplementary Material are presented within the main manuscript.

**Clinical data**

Patients with IBD were recruited to donate faecal samples for microbiome analysis through a speciality IBD clinic. Control subjects were enrolled in study protocol APC055, which was approved by the Clinical Research Ethics Committee of the Cork Teaching Hospitals. All donors completed a questionnaire demonstrating their willingness to partake in the study. Summary details and statistical comparisons of important clinical data are presented in Supplementary Tables 1-3. Briefly, Wilcoxon and Kruskal-Wallis tests assessed a continuous variable against two, or more than two, categorical variables, respectively. Chi-square tests were performed to identify differences in categorical variables, with Fisher’s exact test implemented for small sample sizes. The Shapiro-Wilk test was conducted to evaluate normal distribution of data.


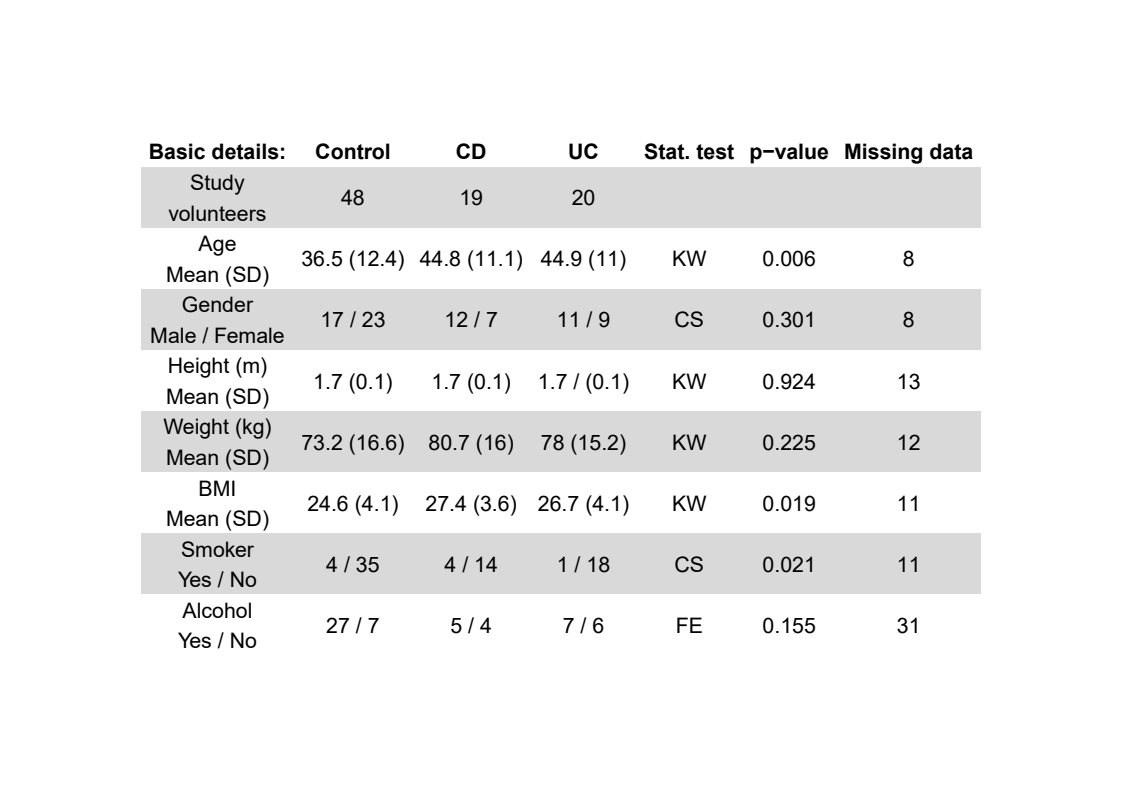
**Supplementary Table 1.** Overview of the basic study volunteer physical and lifestyle data collected. Abbreviations of the pertinent statistical tests are as follows: KW, Kruskal-Wallis; CS, Chi-square; FE, Fisher’s exact.

**Supplementary Table 2.** Overview of the clinical data for patients with CD and UC, describing their disease diagnosis, status, severity, and distribution. Abbreviations for the pertinent statistical tests conducted are as follows: W, Wilcoxon; FE, Fisher’s exact; SW, Shapiro-Wilk.


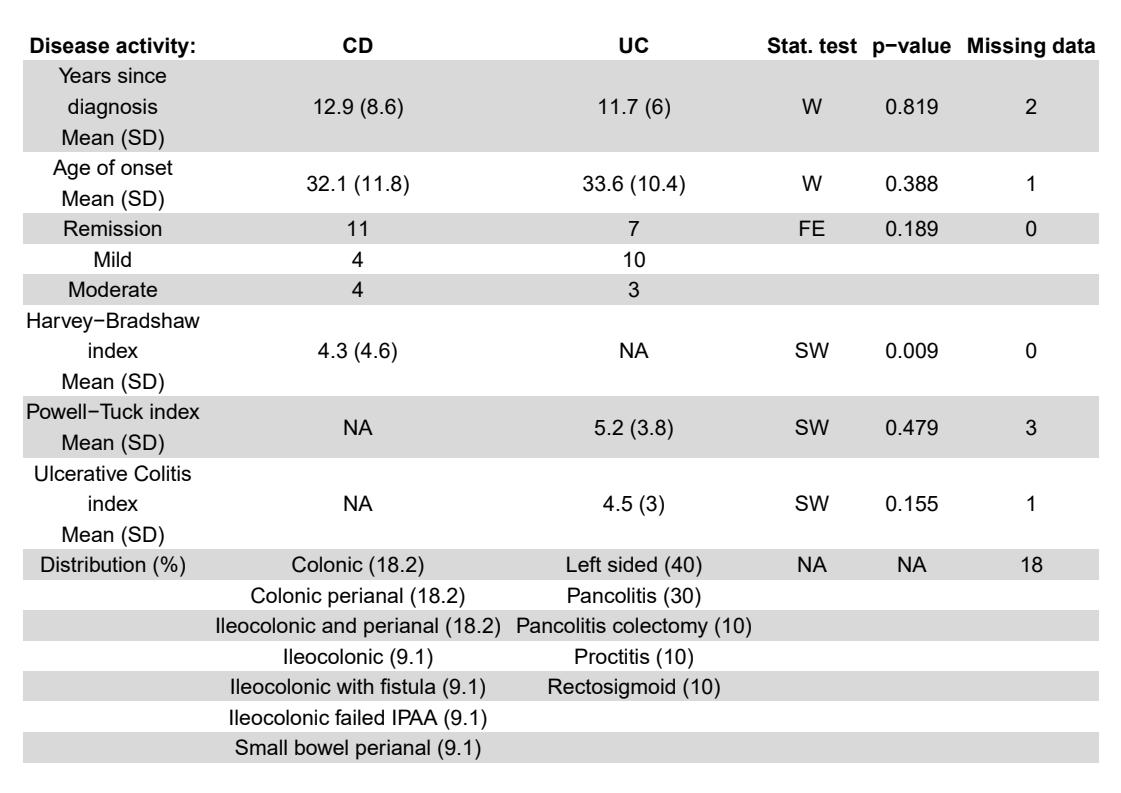

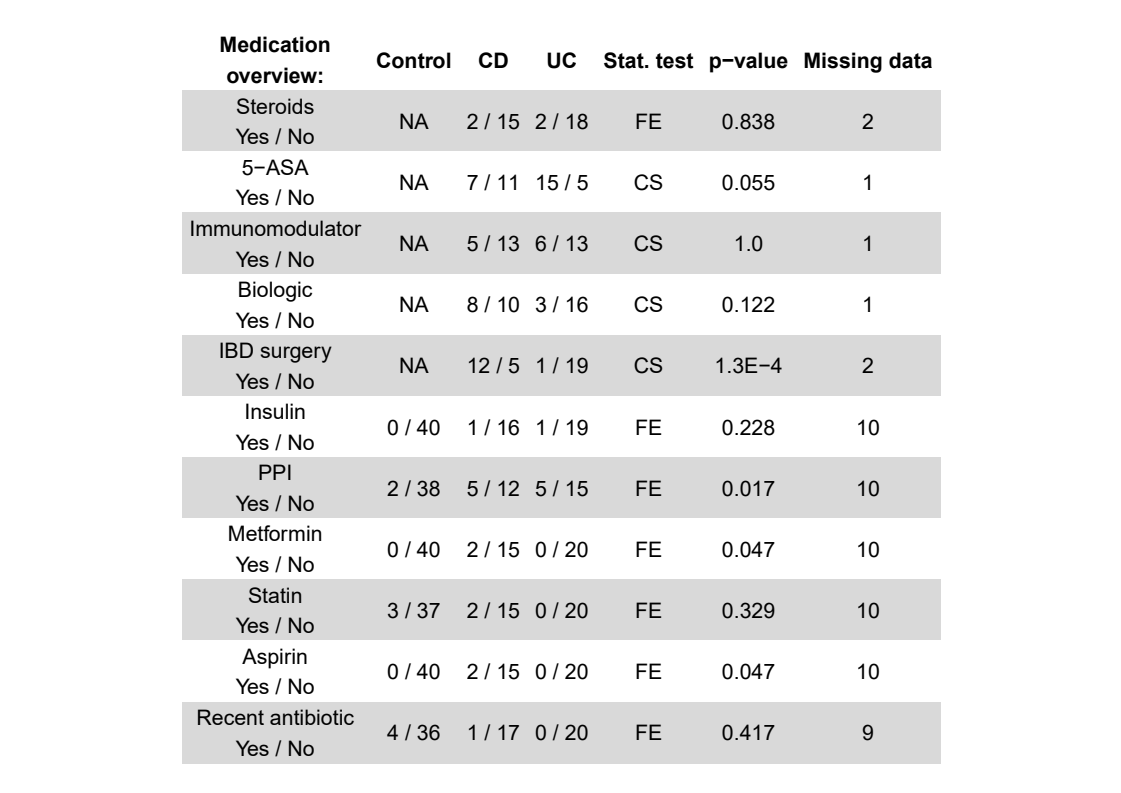
**Supplementary Table 3.** Overview of IBD-specific and generic medications taken by control faecal donors and patients with IBD during enrolment. Abbreviations for the pertinent statistical tests conducted are as follows: FE, Fisher’s exact; CS, Chi-square.

**Building a human-associated plasmidome**:

Bacterial plasmids and plasmidomes have received little attention compared to analyses of bacteria. This is clearly evident when publicly available database sizes are scrutinized. The NCBI RefSeq database (release 203) contained 63,237 curated bacterial genome sequences, but only 5,222 plasmids. This is despite approximately 50% of bacteria carrying plasmids^1^, and mobile genetic elements representing the most diverse components of bacterial strains and microbiomes.

Plasmids with similar replication control mechanisms are typically incapable of residing within the same cell. This is termed plasmid incompatibility. One of the critical steps we employed to identify metagenome assembled plasmids (MAPs) within metagenomic data was the presence of a plasmid replication protein. However, this step introduces a bias as not all plasmids encode their own plasmid replication protein (e.g. plasmid ColE1). Nonetheless, we sought to determine if specific plasmid replication proteins were associated with distinct features (Supplementary Figure 1).


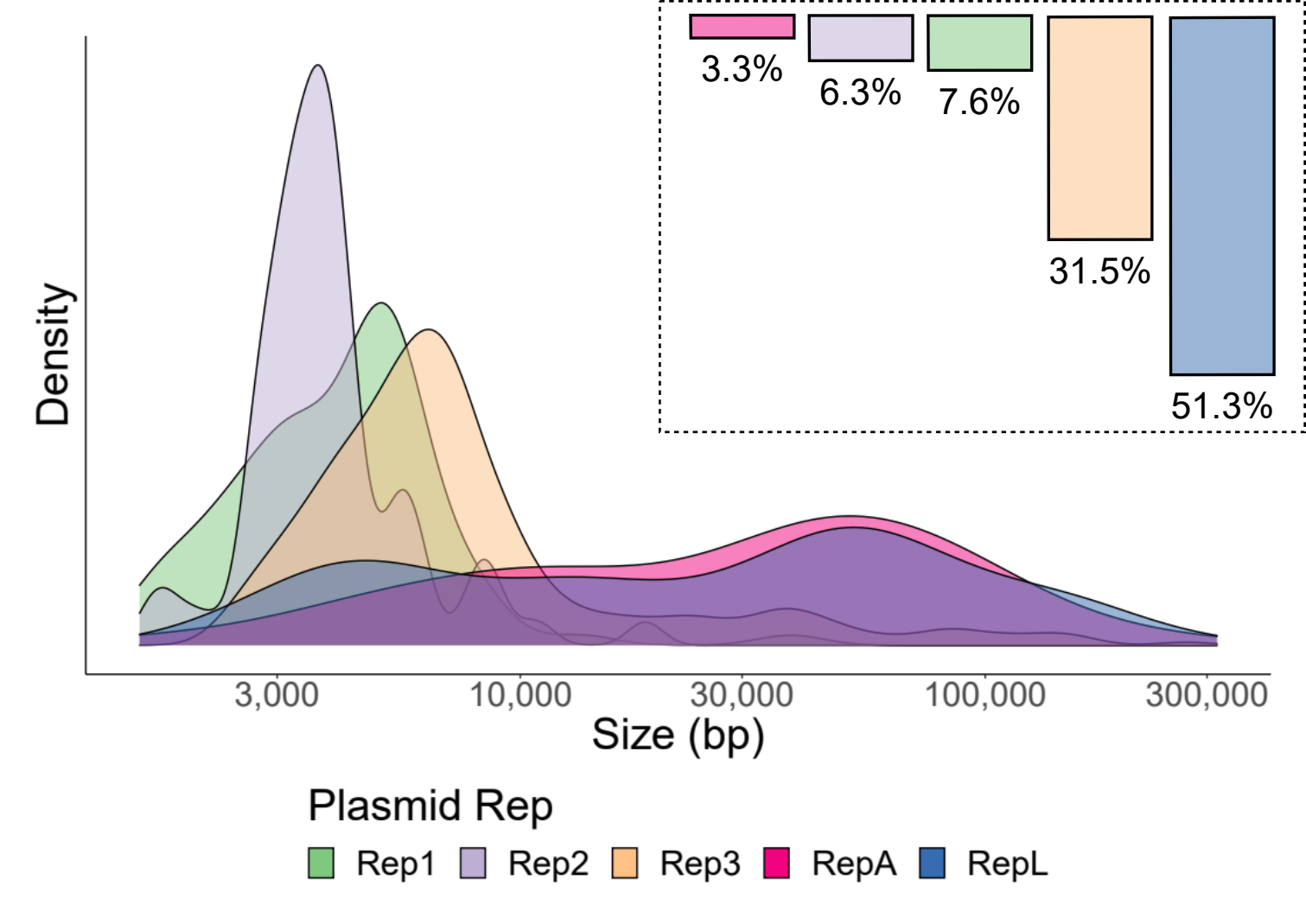
**Supplementary Figure 1.** Size distribution and relative abundance (image inset) of MAPs with specific plasmid replication proteins detected. The results for RepA C-term and RepA N-term were combined (Pfam accessions PF04796 and PF06970, respectively).

**Horizontal gene transfer and antimicrobial resistance:**


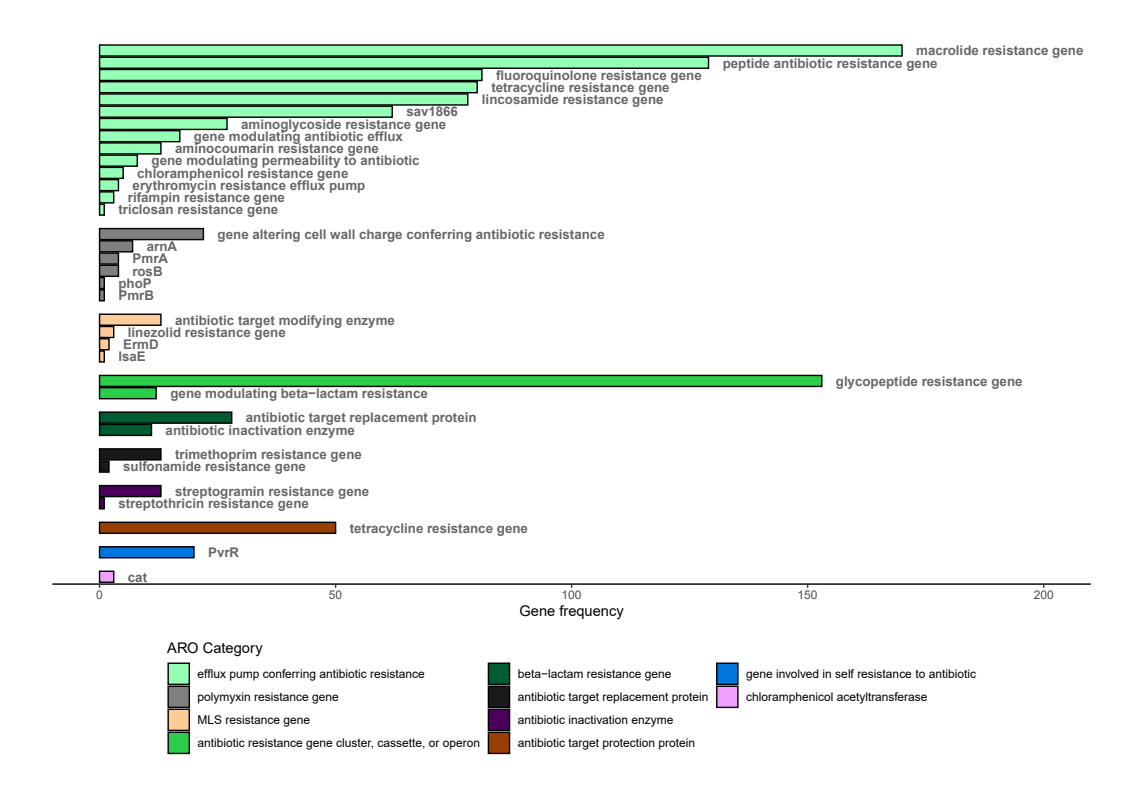
 Plasmids are particularly important in the horizontal gene transfer and rapid mobilization of virulence factors, including antibiotic resistance. The major antibiotic resistance mechanism detected on MAPs based on the result from the comprehensive antibiotic resistance database (CARD) was efflux pumps, followed by target alteration, target protection, and antibiotic inactivation mechanisms. Amongst all AMR genes observed, macrolide, lincosamide, and streptogramin (MLS) resistance genes were particularly abundant, with the *macB* gene the most frequently detected (Supplementary Figure 2).

**Supplementary Figure 2.** Frequency of antibiotic resistance ontology (ARO) grouping of AMR genes detected across the human-associated plasmidome database.

In order to assess the accuracy of predicting the bacterial host of putative plasmids using CRISPR spacers, results were compared with NCBI NT records where a plasmid had a match to that database. While only a limited number of plasmid host predictions could be compared, there were three discrepancies (Supplementary Figure 3). Firstly, NCBI NT recorded several plasmids as belonging to *Bacteroides*, while CRISPR spacers predicted them as either *Bacteroides* or *Parabacteroides*. Secondly, plasmids harboured by *Bacteroides* through CRISPR spacer analysis were recorded by NCBI as multiple Bacteroidetes taxa. Finally, plasmids predicted as *Haemophilus* through CRISPR spacers were listed as *Haemophilus* and *Actinobacillus* by NCBI NT.


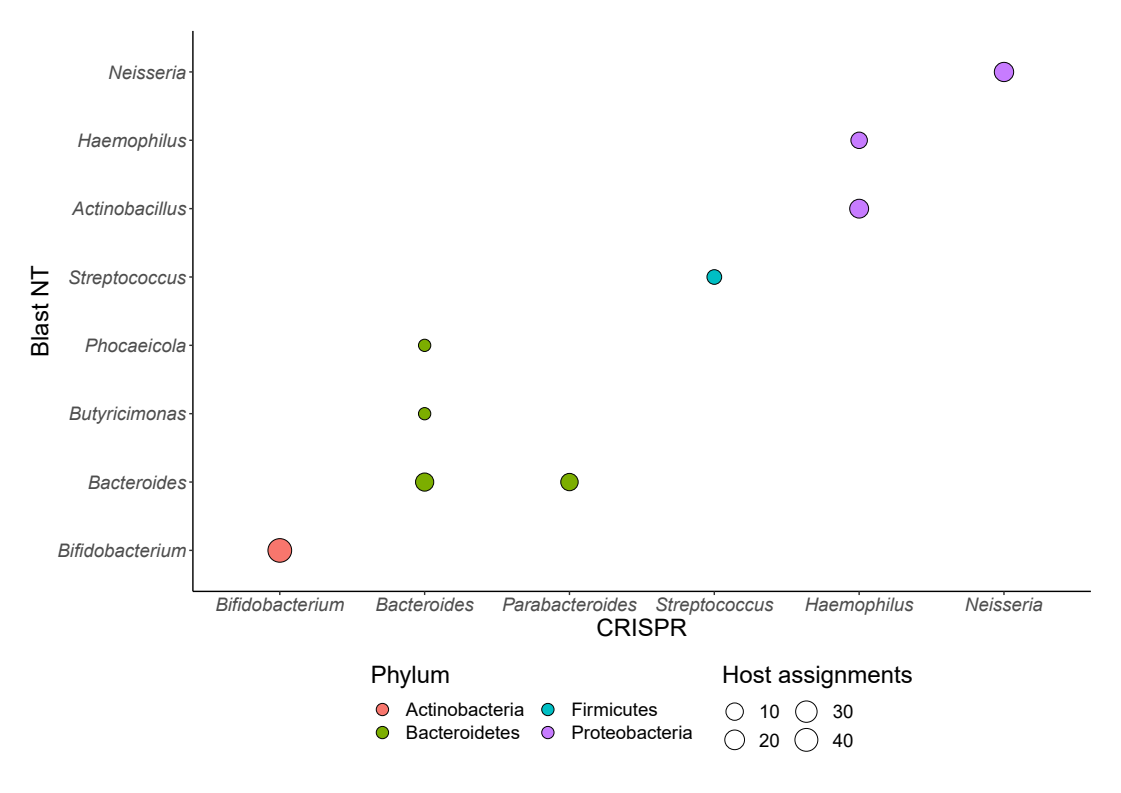
**Supplementary Figure 3.** Assessing putative plasmid host predictions through CRISPR spacers where a plasmid had a homologous plasmid and host bacterium recorded with the NCBI NT database.

**Intra- and inter-sample plasmidome diversity:**


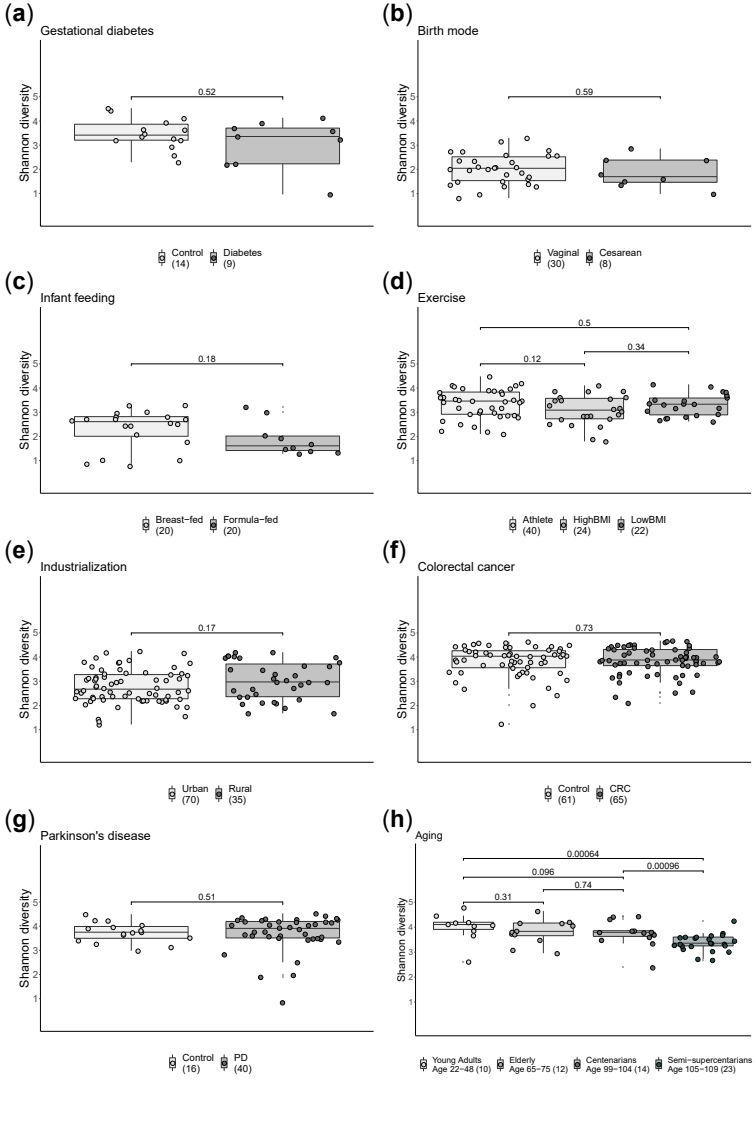
 Diversity analyses of microbiomes are frequently performed to assess if differences are associated with lifestyles, life stages and events. Intra-sample plasmidome α-diversity differences were analysed across multiple publicly available microbiome studies chosen to capture the breadth of human-microbe studies that have been investigated (Supplementary Figure 4). Of conditions investigated, only extreme old age (105-109 years old) was statistically significant relative to the study’s controls. Loss of gut microbiome diversity with age has previously been reported, and is associated with increased frailty and reduced cognitive performance^2^.

**Supplementary Figure 4.** Shannon index α-diversity comparisons of faecal plasmidomes across cohorts analysing various lifestyles, life stages and events. The title of each figure panel indicates the study’s investigative goal. Figure legends show number of samples per cohort (in brackets). Wilcoxon p-values are shown for specific group comparisons.

β-diversity compositional differences were also assessed across lifestyles, life stages and events. Permutational multivariate analysis of variance (PERMANOVA) was assessed for inter-sample Canberra distances used to generate PCoA plots (data not shown). The variances (R^2^) and p-values indicating statistical differences highlights that plasmidomes vary to different degrees across human conditions and are not always significant (Supplementary Table 4).

**Supplementary Table 4.** The number (N) of samples analysed, PERMANOVA variance (R^2^) as a percentage, and p-values for β-diversity compositional differences in faecal plasmidomes across various lifestyles, life stages and events. Statistically significant p-values (<0.05) are in bold. The three IBD studies are as follows: this study’s data; HMP, Human Microbiome Project data^3^; Paediatric CDI, Bushman and team’s infant IBD and CDI data^4^.

| **Study** | **N. samples** | **Variance** | **P-value** |
| --- | --- | --- | --- |
| Pregnancy | 23 | 5.0% | 0.241 |
| Birth mode | 38 | 5.2% | **0.001** |
| Infant feeding | 40 | 7.0% | **0.002** |
| Athletics & BMI | 86 | 2.8% | **0.034** |
| Industrialisation | 105 | 3.4% | **0.001** |
| Colorectal cancer | 126 | 0.9% | 0.135 |
| Parkinson’s disease | 56 | 1.7% | 0.739 |
| Old age | 59 | 6.8% | **0.001** |
| IBD – This study | 87 | 5.2% | **0.001** |
| IBD – HMP | 35 | 6.4% | **0.001** |
| IBD – Paediatric CDI | 147 | 8.3% | **0.001** |
| CDI FMT | 262 | 6.4% | **0.001** |

In order to better understand the β-diversity inter-sample differences associated with faecal plasmidomes, the predicted microbial composition of samples was correlated with the predicted hosts of plasmids. *Fusobacterium*, *Methanobrevibacter*, and *Prevotella* compositions of the overall microbiome and plasmidome strongly correlate, while *Bacteroides*, *Eubacterium*, and *Bifidobacterium* moderately correlate (Supplementary Figure 5). Poor correlations in microbiome and plasmidome specific taxa can be explained as; (i) mobile plasmids may have more than one phylogenetically distant hosts, (ii) the majority of plasmids could not be assigned to a host, and (iii) differences in the recording of bacterial taxa names (e.g. *Clostridium* vs Clostridiales). Nonetheless, the two dimensional ordination of the plasmidome β-diversity matches the separation of the overall microbiome composition. This is similarly in agreement with the current understanding of IBD, where patients experience an overall decrease in bacterial diversity with an increase in specific microbial taxa. For instance, the direction and magnitude of *Prevotella* and *Ruminococcus* associate with
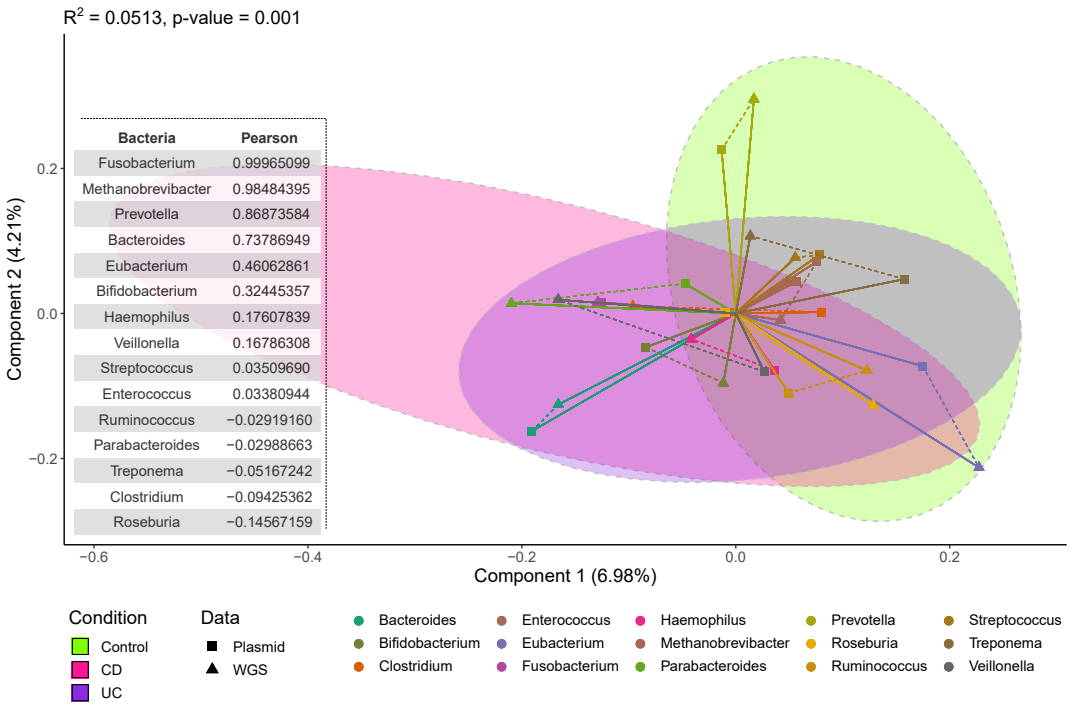
controls, while *Bacteroides* and *Fusobacterium* are associated with IBD.

**Supplementary Figure 5.** Ellipses shown for β-diversity separation of samples by PCoA ordination of Canberra distances. The magnitude and direction of plasmid predicted hosts (square) and whole genome sequencing (WGS) predicted hosts (triangle) from the origin (0,0) are shown as correlations versus PCoA axis 1 and 2. Dotted lines connect specific host predictions from the different data types. The image inset shows the Pearson’s correlation coefficient of WGS taxonomic composition versus plasmid predicted hosts.

Consistent alterations in plasmidome α-diversities were observed across studies that were designed to analyse controls versus patients with IBD and *Clostridioides difficile* infections (CDI). Patients with IBD, both ulcerative colitis (UC) and Crohn’s disease (CD), and CDI are regularly reported as possessing an altered gut microbiome with reduced microbial diversity. In addition to the IBD microbiome sequencing results presented in this study, paediatric IBD data of Bushman *et al.* ^4^ and Fraser-Liggett ^3^ are presented (Supplementary Figure 6).


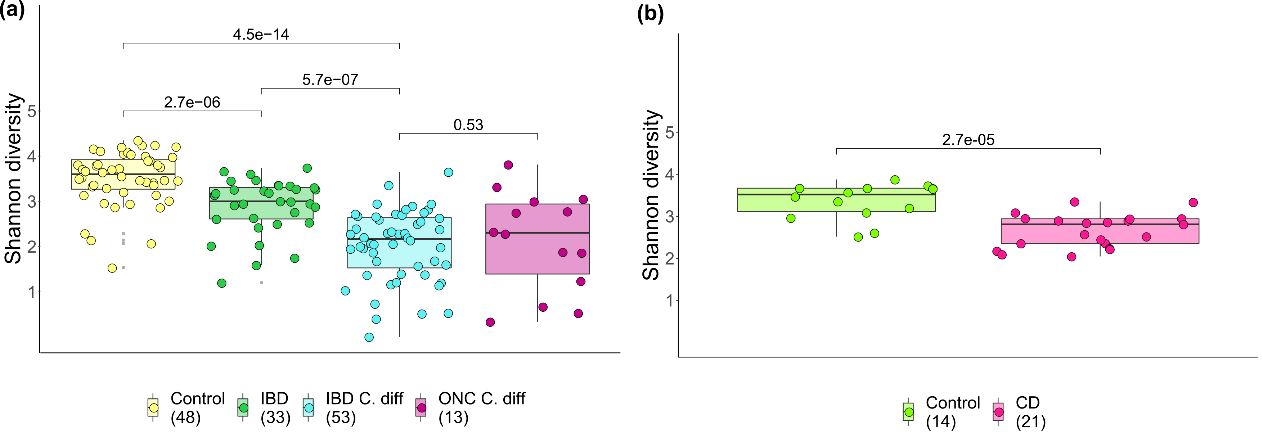


**Supplementary Figure 6.** Shannon index α-diversity comparisons of faecal plasmidomes of **(a)** control children versus paediatric IBD, paediatric IBD with *C. difficile*, and paediatric cancer (ONC) with *C. difficile*, and **(b)** Human Microbiome Project (HMP) control adults versus patients with Crohn’s disease (CD). Figure legends show number of samples per cohort (in brackets). Wilcoxon p-values are shown for specific group comparisons.

Sample diversity is dependent on the number of species present (richness) and the population size of each species present (evenness). Therefore, to further understand the α-diversity differences observed between control and IBD faecal plasmidomes, richness and evenness measures were assessed. For individuals with CD or UC, both the abundance of plasmids and their representation within plasmidomes are decreased in IBD (Supplementary Figure 7).


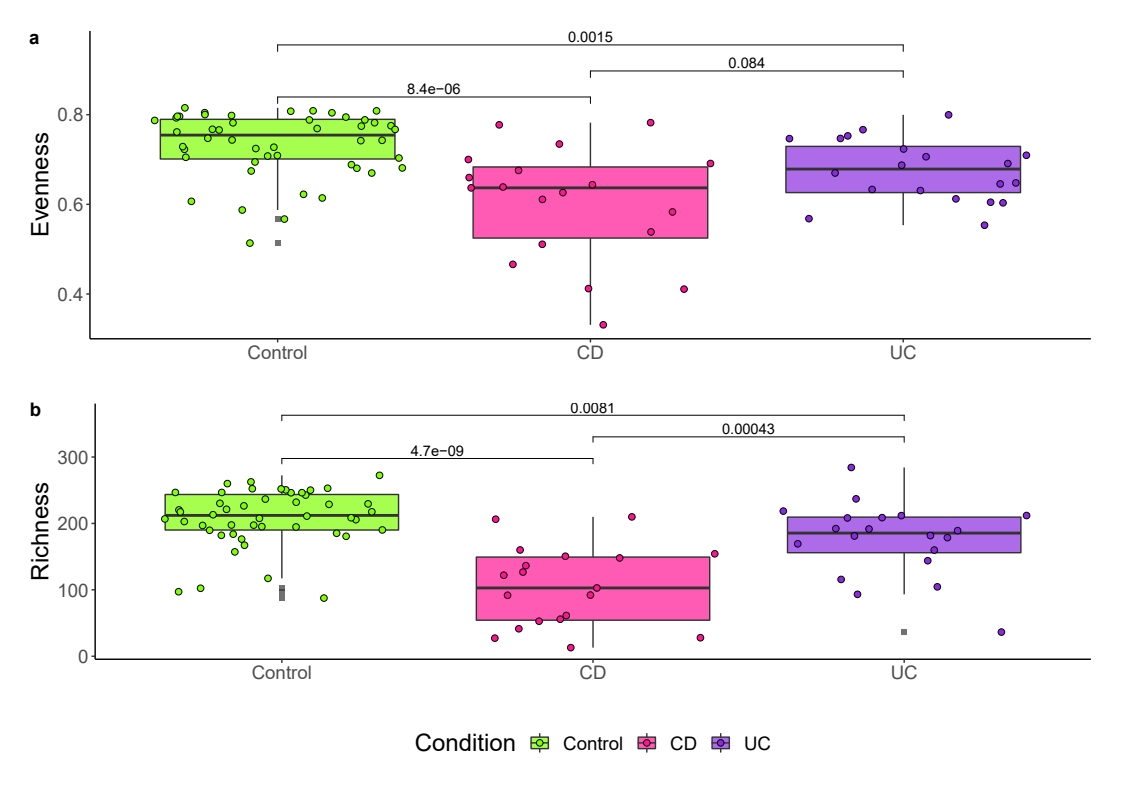
**Supplementary Figure 7.** Comparison of α-diversity evenness and richness measures for control, CD, and UC plasmidomes. Wilcoxon statistical test p-values are shown for specific group comparisons.

To investigate the effect of faecal microbiota transplants (FMTs) on patients with IBD and CDI, the FMT study data was further analysed. Of note, four patients with IBD were treated for CDI using FMT. All patients demonstrated increased plasmidome α-diversities up to 12 weeks after FMT (Supplementary Figure 8).

**
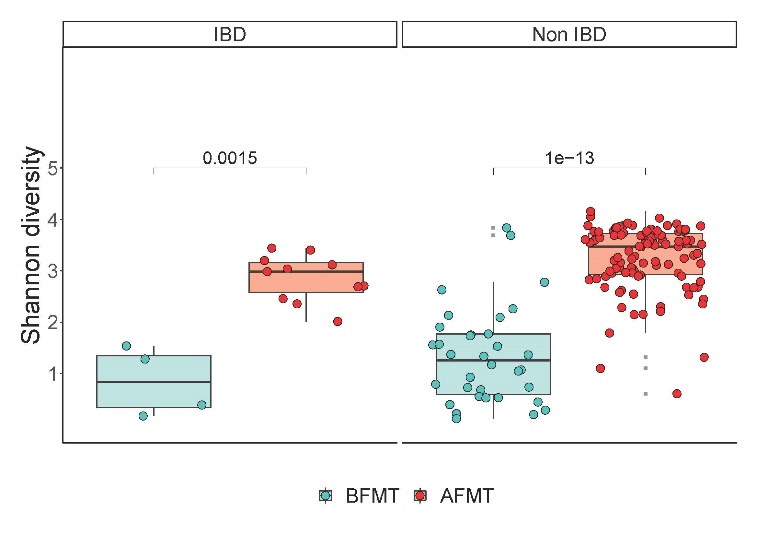
**

**Supplementary Figure 8.** The α-diversities of plasmidomes of patients with CDI also with IBD and without IBD before and after FMT. Wilcoxon statistical test p-values are shown for specific group comparisons.

**Plasmidome functions**

Due to the large disparities in plasmid lengths, our analyses avoided using relative abundances. As an example, if a 1kb and 100,000kb plasmid within a sample each recruiting 1000 reads, they would have the same relative abundance. However, intuitively, if these plasmids were present in the same abundance during sequencing, the longer sequence would recruit far more reads. Therefore, we adopted the reads per kilobase per million reads (RPKM) procedure commonly utilised in transcriptomic studies to normalise read counts. Furthermore, when investigating plasmid-encoded functional differences, the counts matrix was normalised as reads per gene length per million reads per subject. The abundance of plasmid-encoded functional predictions were subsequently aggregated per person, and assessed for Wilcoxon test statistical significance between individuals categorised in the control, CD, and UC cohorts. As patients with IBD, and particular those with CD, have diminished plasmidomes, the general trend was for decreased plasmid functions associated with IBD. Statistically significant plasmid-encoded functional differences after Bonferroni correction were between control and patients with CD, featuring a variety of cellular functions (Supplementary Table 5).

**Supplementary Table 5.** All statistically significant plasmid-encoded functional differences between control, CD, and UC cohort combinations. The abundance of plasmid-encoded functions per person was normalised as the number of reads per gene length per million reads per sample. The abundance of plasmid-encoded functions within the total plasmidome was subsequently aggregated per person before Wilcoxon paired statistical comparison. P-values shown are after Bonferroni correction.


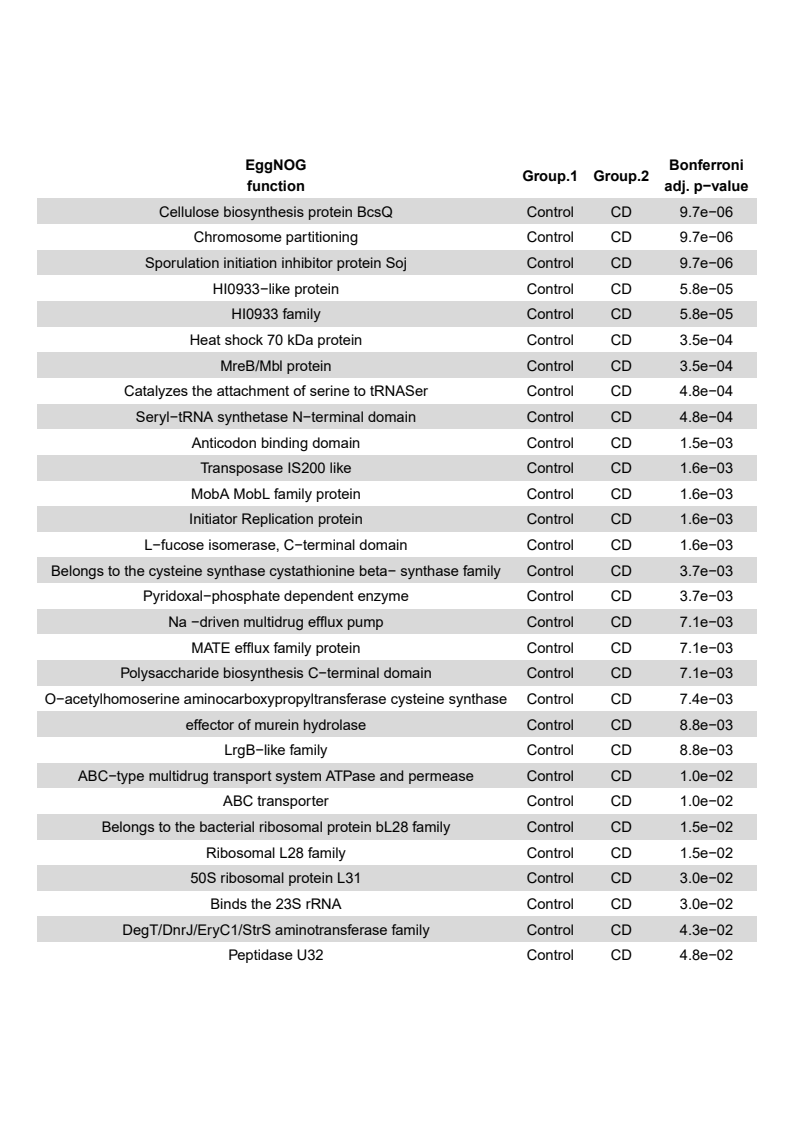


**Supplementary references**

1. Clark, D. P., Pazdernik, N. J. & McGehee, M. R. Plasmids. in *Molecular Biology 712–748* (Elsevier, 2019). doi:10.1016/B978-0-12-813288-3.00023-9.

2. Claesson, M. J. *et al.* Gut microbiota composition correlates with diet and health in the elderly. *Nature* **488**, 178 (2012).

3. Fraser-Liggett, C. Metagenomic Analysis of the Structure and Function of the Human Gut Microbiota in Crohn’s Disease. *Nat Preced [Internet* (2010).

4. Bushman, F. D. Multi-omic Analysis of the Interaction between Clostridioides difficile Infection and Pediatric Inflammatory Bowel Disease. *Cell Host Microbe* **28**, 422-433 7 (2020).
